# A randomized phase 1 single-dose polysomnography study of ASP8062, a GABA_B_ receptor positive allosteric modulator

**DOI:** 10.1007/s00213-020-05738-y

**Published:** 2021-01-12

**Authors:** Mark Walzer, Ruishan Wu, Maha Ahmad, Jon Freeman, Gary Zammit, Gerard J. Marek

**Affiliations:** 1 Astellas Pharma Global Development, Inc., One Astellas Way, Northbrook, IL 60062 USA; 2 Clinilabs Drug Development Corporation, New York, USA

**Keywords:** Polysomnography, GABA_B_ receptor positive allosteric modulator, Safety/tolerability, Pharmacokinetics, Pharmacodynamics, Slow-wave sleep, ASP8062, Healthy volunteer

## Abstract

**Rationale:**

Previous research suggests that sleep polysomnography and EEG endpoints can be used to assess GABAergic activity; however, the impact of GABA_B_ receptor positive allosteric modulators on sleep endpoints remains unclear.

**Objectives:**

This phase 1 study compared a single dose of ASP8062 (35 mg or 70 mg), a GABA_B_ receptor positive allosteric modulator, with placebo and paroxetine (40 mg).

**Methods:**

Healthy adult volunteers were randomized to four treatments (35 mg ASP8062, 70 mg ASP8062, paroxetine 40 mg, or matching placebo), each separated by a 14-day washout. Primary endpoints obtained by polysomnography were time in stage N3 or SWS and time in rapid eye movement (REM) sleep. Secondary endpoints included impact on sleep stages and electroencephalography parameters, pharmacokinetics, nighttime growth hormone (GH), and safety/tolerability.

**Results:**

In 20 randomized volunteers, ASP8062 led to a significant and seemingly dose-dependent increase in SWS over the entire night; this increase was mainly observed during the first third of the night. ASP8062 did not impact time in REM sleep. Paroxetine had no effect on SWS but produced a significant reduction in time spent in REM sleep. A dose-dependent trend in increased GH release was also observed with ASP8062. Headache and nausea were the most commonly reported treatment-emergent adverse events (TEAEs) for ASP8062; most TEAEs were mild in severity.

**Conclusions:**

Single-dose ASP8062 (35 and 70 mg) appeared to result in CNS penetration and enhanced GABAergic activity as measured by increases in slow-wave sleep and growth hormone release.

**Supplementary Information:**

The online version contains supplementary material available at 10.1007/s00213-020-05738-y.

## Introduction

Polysomnography (PSG), considered the gold standard for evaluating patients with sleep disorders, is also an ideal tool or biomarker for assessing drugs with central nervous system (CNS) pharmacology because it allows simultaneous and continuous measurement of multiple physiologic parameters during sleep and can be used in healthy volunteers (Rundo and Downey 2019; Kushida et al. 2005; Kapur et al. 2017; Matheson et al. 2007). PSG can be used in CNS-related studies to determine a variety of endpoints, including sleep latency, wake after sleep onset, sleep efficiency (total sleep time divided by total recording time), percentage of each sleep stage, and arousals from sleep both in animal studies and human trials (Rundo and Downey 2019). Combined with assessment of other electroencephalographic (EEG) sleep spectra endpoints, PSG studies allow for comprehensive monitoring of treatment effects on sleep stage and CNS activity after administration of an investigational medication (Rundo and Downey 2019; Matheson et al. 2007).

The G protein–coupled receptor for γ-aminobutyric acid (GABA), the metabotropic GABA_B_ receptor, is purported to play an important role in maintaining sleep, particularly in sustaining sleep through the night, and is suggested to have anti-nociceptive effects, anxiolytic activity, and suppressive effects on drug-seeking behavior (Gmeiner et al. 2013; Pin and Prezeau 2007).

Among these, the effects of enhancing GABAergic transmission in maintaining sleep by increasing slow-wave sleep (SWS) is particularly compelling (Gottesmann 2002; Bellesi et al. 2014). Previous PSG studies of GABA_B_ agonists (e.g., baclofen and sodium oxybate) have consistently shown an increase in N3 (SWS) and delta and theta wave activity in both patients with narcolepsy and other disorders with altered sleep-associated symptoms as well as in healthy volunteers. These data are less conclusive regarding the impact of GABA_B_ agonists on nighttime release of growth hormone (GH) as well as the impact on rapid eye movement (REM) and other stages of sleep (Van Cauter et al. 1997; Vienne et al. 2012; Dornbierer et al. 2019). These earlier studies created the basis for assessing CNS pharmacological activity of a GABA_B_ using PSG and EEG endpoints during sleep. Despite the effects of GABA_B_ agonists on sleep, clinical use of these compounds is limited because of poor brain penetration, tolerance issues, and complications, including altered mental status, agitation, drug diversion, seizures, sleep paralysis/coma, and the potential use as a facilitator of date rape (Russell et al. 2011; Dario and Tomei 2004; Chin et al. 1998). The tolerance issues and complications associated with baclofen and sodium oxybate demonstrated in these studies highlight the need for agents that can enhance GABA_B_ activity without producing undesirable side effects. The use of GABA_B_ receptor positive allosteric modulators (PAMs) may represent such an alternative approach (McCarson and Enna 2014). GABA_B_ receptor PAMs enhance the effects of GABA and, consequently, enhance receptor activity in a physiological manner, with a lower risk of tolerance issues and undesirable side effects than those seen with GABA_B_ agonists (Pin and Prezeau 2007; Ong and Kerr 2005; Bowery 2006).

ASP8062 is a novel, orally active GABA_B_ receptor PAM, previously studied in vitro and also in vivo to evaluate its effects on the sleep/wake cycle, EEG during sleep stages, and motor coordination in rats (Murai et al. 2019). In these studies, ASP8062 significantly decreased REM sleep, increased the power of delta waves during non-REM sleep, and significantly decreased the frequency of sleep interruptions. Therefore, ASP8062 was evaluated in a phase 1 single ascending oral dose study in healthy males administered doses of up to 70 mg (8062-CL-0001) and in a multiple ascending oral dose study in healthy male and female volunteers (8062-CL-0002) administered doses of up to 30 mg or placebo for 14 consecutive days (Murai et al. 2019; Walzer et al. 2020). ASP8062 had a tolerable safety profile at all doses after single and multiple administrations, and most treatment-emergent adverse events (TEAEs) were considered mild in severity. The most common TEAEs across both studies were dizziness and headache. No deaths, serious adverse events (AEs), or TEAEs leading to withdrawal of treatment occurred throughout either study (Walzer et al. 2020). After a single dose of 30-mg (*n* = 6) or 70-mg (*n* = 6) ASP8062, the median time to peak plasma concentration (*T*_max_) was 4 and 2.5 h, respectively. However, absorption was very rapid in some subjects, with *T*_max_ less than 1 h, and slow in other subjects, with *T*_max_ delayed to 8 h. The distribution of ASP8062 in the cerebrospinal fluid (CSF) after a single dose was also rapid, and similar to plasma, but slightly delayed; median *T*_max_ times of 5 and 3 h were observed in the CSF following 10-mg (*n* = 4) or 70-mg (*n* = 4) single doses, respectively. Concentrations of ASP8062 persisted in CSF over the full 24-h sampling schedule (Walzer et al. 2020).

The current randomized, double-blind, crossover PSG study evaluated the CNS pharmacodynamic effects, pharmacokinetic profile, and tolerability of two doses of ASP8062 in healthy volunteers in comparison with placebo and the selective serotonin reuptake inhibitor (SSRI), paroxetine (active control). In addition, previous research has shown that large pulses of GH secretion occur simultaneously during the first slow-wave period in healthy volunteers and the amount of GH release is proportional to the duration of SWS (Van Cauter et al. 1997). Therefore, GH secretion was measured as a confirmatory biomarker in this study. Paroxetine was used as the positive control because of its demonstrated effects on REM sleep both in healthy volunteers and in patients with major depressive disorder (Winokur et al. 2001; Steiger and Pawlowski 2019). This is a key study in the clinical development of ASP8062 owing to the predicted increase in SWS. Therefore, the goal was to provide indirect evidence suggesting CNS penetration for drug exposures observed with these ASP8062 doses (Gmeiner et al. 2013; Russell et al. 2011; Spaeth et al. 2012; Anaclet and Fuller 2017; Luppi et al. 2017).

## Methods

### Study design

In this double-blind, placebo- and active-controlled, single-dose, 4-way crossover PSG study, eligible healthy volunteers were randomly assigned to receive single oral doses of 35-mg ASP8062, 70-mg ASP8062, paroxetine 40 mg, or matching placebo 2 h before planned bedtime (Table 1). The single 70-mg dose of ASP8062 was selected to match exposures to a 30-mg QD dose, which was the highest dose administered in the 14-day multiple ascending dose study (Walzer et al. 2020).

**Table 1:** Treatment sequences, treatment periods, and doses

Treatment Sequence	Treatment Period and Doses			
	1	2	3	4
1	ASP8062 70 mg	Placebo	ASP8062 35 mg	Paroxetine 40 mg
2	ASP8062 35 mg	ASP8062 70 mg	Paroxetine 40 mg	Placebo
3	Paroxetine 40 mg	ASP8062 35 mg	Placebo	ASP8062 70 mg
4	Placebo	Paroxetine 40 mg	ASP8062 70 mg	ASP8062 35 mg

All individuals who were selected to participate in this study were required to maintain a sleep diary for at least 28 days prior to enrollment in order to determine their median habitual bedtime. The healthy volunteers were admitted to the clinical unit 1 day before the four treatment periods (day − 1), each of which lasted for a duration of 5 days and 4 nights. PSG assessment during the clinical unit habituation (day − 1) only occurred during each subject’s period 1. The four treatment periods were separated by a washout of at least 14 days. After randomization, the healthy volunteers received their assigned treatment on day 1, followed by a PSG assessment during sleep and a 60-h in-house blood sampling period. Food and liquids (except water) were restricted from 3 h prior to dosing until 8 h after dosing. The healthy volunteers were discharged from each period on day 4.

The ethical, scientific, and medical appropriateness of this study was evaluated by an institutional review board before the start of the study. The study was conducted in accordance with the ethical principles of the Declaration of Helsinki, Good Clinical Practice, International Council for Harmonization guidelines, and applicable laws and regulations. All healthy volunteers were required to provide an institutional review board—approved written informed consent—and to sign a Health Insurance Portability and Accountability Act authorization form.

### Study subjects

Healthy men and women between 18 and 55 years of age with a body mass index between 18.5 and 32 kg/m^2^ were eligible for inclusion in this study. Subjects were required to follow a typical bedtime between 21:00 and 01:00, with a typical total sleep time (TST) of at least 7 h.

Subjects were excluded from the study if they had known or suspected hypersensitivity to ASP8062 or paroxetine, or contraindication to paroxetine. Subjects were also excluded if they had a recent history of sleep disorder, such as insomnia, sleep apnea, narcolepsy, restless legs syndrome, periodic limb movement disorder, circadian rhythm disorder, or other clinically significant sleep disturbance, as determined by a board-certified sleep specialist.

### Study outcomes

PSG consisted of an 8-h (960 epochs) assessment during which EEG, electrooculographic, ECG, and submental electromyographic activity were recorded according to methods consistent with the standards of the American Academy of Sleep Medicine. PSG was manually scored by an experienced sleep technician using American Academy of Sleep Medicine criteria. In order to simplify reporting of the results, the waveforms were grouped into waveforms of 1 to 12 Hz (frequency bands delta (1 to 3.5 Hz), theta (3.5 to 8 Hz), and alpha (8 to 12 Hz)) and waveforms of > 12 Hz (frequency bands sigma (12 to 16 Hz), beta 1 (16 to 24 Hz), beta 2 (24 to 32 Hz), and gamma (32 to 48 Hz)). The recordings were started at the subjects’ median habitual bedtime, ± 30 min, on the day of dosing within each treatment period. Machine calibrations and bio-calibrations were performed approximately 30 min and 15 min before lights-off, respectively. Following dosing, the subjects were required to remain in bed for the duration of the 8-h PSG recording period, except for interruptions to use the restroom. PSG primary endpoints included time in stage N3, or SWS, and time in REM sleep. Other PSG endpoints assessed included total minutes of REM sleep, total minutes of stage N1 sleep, total minutes of stage N2 sleep, latency to persistent sleep in minutes, latency to REM sleep in minutes, latency to each REM sleep period in minutes, cumulative REM sleep time in minutes-per-night recording time, wakefulness after sleep onset in minutes, number of awakenings, total sleep time in minutes, and sleep efficiency power spectral EEG analysis. Quantitative EEG results were reported in both absolute and relative power for both SWS and REM sleep. Endpoints were measured in 30-s epochs.

In this study, PSG was performed in accordance with the American Academy of Sleep Medicine’s guidelines using an Alice 6 LDxS (Philips Respironics, Murrysville, PA) or Embletta MPR PG (Natus Medical, Pleasanton, CA) device. A trained sleep technician collected PSG data and analyzed them using the acquisition program GRASS-GAMMA version 4.4; staging data was imported into Stellate HARMONIE 5.0 digital system (Stellate Systems Inc., Quebec, Canada) to analyze the EEG.

Blood samples were collected predose and at 45 min, 1 h, 1 h 30 min, 2, 3, 4, 8, 10, 12, 24, 48, and 60 h postdose during sleep using an indwelling catheter to assess the pharmacokinetic profile of ASP8062 and paroxetine. Measurements of area under the concentration-time curve from the time of dosing to the last measurable concentration (AUC_last_), AUC from the time of dosing to 10 h postdose (AUC_10_), AUC from the time of dosing to 24 h postdose (AUC_24_), maximum concentration (*C*_max_), and time of maximum concentration (*T*_max_) were also included. Blood samples were also collected to assess changes in GH levels. Safety assessments included monitoring of AEs, full spectrum of suicidality (suicidal ideation, intensity of ideation, suicidal behavior, and actual attempts) based on the Columbia-Suicide Severity Rating Scale, self-evaluation of mood based on 16 dimensions using the Bond-Lader Visual Analogue Mood Scales, and evaluation of the cognitive safety profile (psychomotor speed, sustained attention, episodic memory, recognition memory, working memory, and executive function) of ASP8062 using a comprehensive cognitive battery assessment.

### Statistical analysis

Up to a total of 20 subjects were randomized in this clinical study. Subjects were assigned to one of four treatment sequences with an equal number of subjects in each treatment sequence. PSG and EEG endpoints were analyzed using an analysis of variance model (ANOVA), including model terms for sequence, period, and treatment as fixed effects and subject nested within sequence as a random effect. Nominal *P* value < 0.1 based on a 2-sided test without multiplicity adjustment was considered statistically significant. Point estimate and 90% confidence interval, along with *P* value, were used for decision-making. Primary PSG endpoints (of SWS (stage N3) and total amount of REM sleep) were assessed over the total night of sleep. Additionally, the night was divided into thirds to explore the primary assessments within shorter periods. The pharmacokinetic and pharmacodynamic data were summarized using descriptive statistics. In terms of safety, all AEs were coded using the Medical Dictionary for Regulatory Activities version 18.0. They were presented as number and percentage of subjects with TEAEs, drug-related TEAEs, TEAEs leading to treatment withdrawal, and drug-related TEAEs leading to treatment withdrawal. A drug-related TEAE was defined as any TEAE with a possible or probable relationship to study drug as assessed by the investigator in a blinded manner.

## Results

### Subject disposition and baseline demographics

Twenty healthy volunteers were randomly assigned to each of four treatment sequences **(**Table 1**)**. Of the 20 healthy volunteers, 19 completed all four treatment periods; one female was lost to follow-up on day 4 after receiving a single oral dose of 70-mg ASP8062 on day 1 of period 1. Healthy volunteers ranged in age from 25 to 54 years, 70% were male, and 70% were Black or African American (Table 2). With respect to concomitant medications, four volunteers used the permitted medication acetaminophen concomitantly to treat the TEAEs of headache, dysmenorrhea, and ear pain.

**Table 2:** Demographic and baseline characteristics

Characteristic	Treatment Sequence				Total (*N* = 20)
	1 (*n* = 5)	2 (*n* = 5)	3 (*n* = 5)	4 (*n* = 5)	
Age, median years (range)	35.0 (29–51)	31.0 (25–32)	42.0 (25–54)	44.0 (31–47)	33.5 (25–54)
Sex, *n* (%)					
Male	3 (60.0)	5 (100.0)	3 (60.0)	3 (60.0)	14 (70.0)
Female	2 (40.0)	0 (0.0)	2 (40.0)	2 (40.0)	6 (30.0)
Race					
White	1 (20.0)	0 (0.0)	2 (40.0)	1 (20.0)	4 (20.0)
African American	3 (60.0)	5 (100.0)	3 (60.0)	3 (60.0)	14 (70.0)
Other	1 (20.0)	0 (0.0)	0 (0.0)	1 (20.0)	2 (10.0)
Body mass index, median kg/m^2^ (range)	28.60 (20.7–31.8)	24.84 (19.5–28.2)	28.75 (23.0–30.7)	31.16 (28.8–31.5)	28.68 (19.5–31.8)

### Polysomnography

**Fig. 1:**
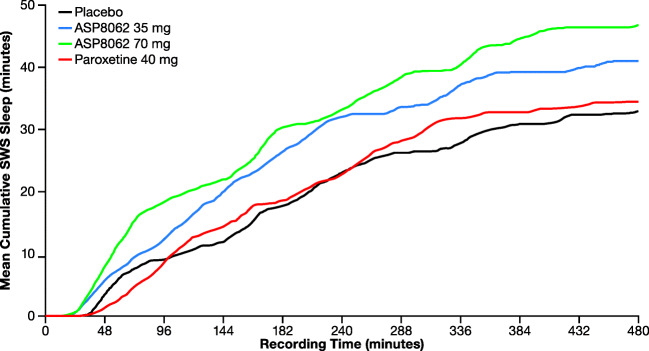
Mean cumulative slow-wave sleep (stage N3) time by time profiles and treatment groups (polysomnography analysis set). Treatment with ASP8062 resulted in a significant increase in the time spent in SWS (stage N3). The least squares mean through the night was 8.18 min greater with 35-mg ASP8062 and 12.68 min greater with 70-mg ASP8062 compared with placebo. This increase recorded in SWS over the entire night with 70- mg ASP8062 and 35-mg ASP8062 was statistically significant compared with placebo (*P* = 0.005 and *P* = 0.067, respectively). Abbreviation: SWS, slow-wave sleep

Treatment with ASP8062 resulted in a significant increase in the time spent in SWS (stage N3). The least squares mean through the night was 8.18 min greater with 35-mg ASP8062 and 12.68 min greater with 70-mg ASP8062 compared with placebo (Table 3**,** Fig. 1). This increase recorded in SWS over the entire night with 70-mg and 35-mg ASP8062 was statistically significant compared with placebo (*P* = 0.005 and *P* = 0.067, respectively). During the first third of the night, the effect on SWS was significantly higher by 8.28 min (35-mg dose) and by 9.11 min (70-mg dose) compared with placebo. No significant differences were observed during the second or last third of the night. The cumulative SWS data are consistent with a dose-dependent increase in SWS throughout the night, although only two doses were tested. Paroxetine had no significant effect on SWS, as expected based on prior data from PSG studies.

**Table 3:** Statistical analysis of SWS (stage N3) and REM polysomnography by recording duration and treatment group (PSG analysis set)

LSM difference from placebo (90% CI)	ASP8062 35 mg (*n* = 19)	ASP8062 70 mg (*n* = 20)	Paroxetine 40 mg (*n* = 19)
Entire night, min			
SWS (stage N3)	8.18 (0.86, 15.51)*	12.68 (5.37, 19.99)^‡^	1.32 (− 6.01, 8.65)
REM	2.02 (− 7.04, 11.08)	6.15 (− 2.81, 15.12)	− 26.92 (− 35.98, − 17.86)^§^
First third of night, min			
SWS (stage N3)	8.28 (2.48, 14.08)^†^	9.11 (3.32, 14.89)^†^	2.21 (− 3.59, 8.01)
REM	4.55 (0.00, 9.10)*	2.18 (− 2.31, 6.68)	− 2.40 (− 6.95, 2.15)
Second third of night, min			
SWS (stage N3)	0.14 (− 6.31, 6.59)	2.33 (− 4.07, 8.73)	2.07 (− 4.38, 8.52)
REM	− 3.35 (− 8.70, 2.00)	− 1.46 (− 6.76, 3.83)	− 13.51 (− 18.85, − 8.16)^§^
Last third of night, min			
SWS (stage N3)	− 0.23 (− 4.07, 3.61)	1.41 (− 2.41, 5.22)	− 2.96 (− 6.80, 0.89)
REM	0.81 (− 4.66, 6.28)	5.46 (0.04, 10.87)*	− 11.01 (− 16.48, − 5.54)^§^

*P ≤ 0.10. ^†^*P* ≤ 0.05. ^‡^*P* ≤ 0.01. ^§^*P* ≤ 0.001

*CI* confidence interval, *LSM* least squares mean, *PSG* polysomnography, *REM* rapid eye movement, *SWS* slow-wave sleep

**Fig. 2:**
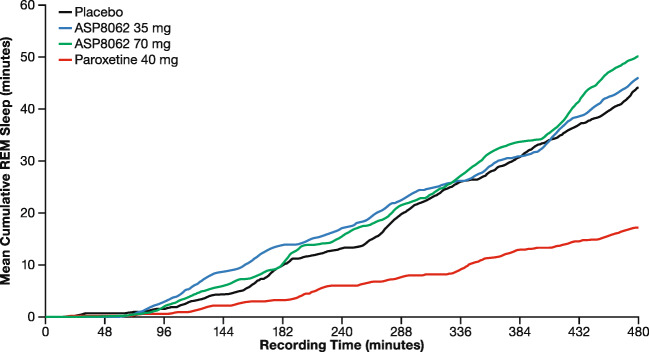
Mean cumulative rapid eye movement sleep time by elapsed time profiles and treatment group (polysomnography analysis set). No consistent or dose-related effect of ASP8062 was observed for time spent in REM sleep compared with placebo. However, paroxetine as a positive control produced a significant reduction in REM sleep (− 8.25%, *P* < 0.001) compared with placebo throughout the night. Abbreviation: REM, rapid eye movement

No consistent or dose-related effect of ASP8062 was observed on time spent in REM sleep compared with placebo (Table 3**,** Fig. 2). However, paroxetine as a positive control produced a significant reduction in REM sleep (− 8.25%, *P* < 0.001) compared with placebo throughout the night, as expected based on prior data.

Treatment with 70-mg ASP8062 reduced stage N1 sleep at multiple time points, and there was a significant decrease in duration and percentage of N1 sleep over the entire night (Table 4). Thirty-five milligrams ASP8062 showed a similar trend with respect to stage N1 sleep time, but the changes did not reach statistical significance. A reduction in sleep efficiency was recorded with paroxetine compared with placebo, but not with ASP8062. No significant effects or obvious trends were seen in sleep efficiency or other PSG parameters between placebo and the 35-mg ASP8062 or 70-mg ASP8062 treatment groups. The paroxetine 40-mg dose group had significantly lower sleep efficiency compared with placebo or ASP8062 dose groups, which was driven by a significant decrease (− 34.48 min [*P* = 0.067]) in TST over the whole night.

**Table 4:** Statistical analysis of polysomnography of other sleep stages by recording duration and treatment group (PSG analysis set)

LSM difference from placebo, entire night (90% CI)	ASP8062 35 mg (*n* = 19)	ASP8062 70 mg (*n* = 20)	Paroxetine 40 mg (*n* = 19)
Stage N1 sleep time, min	− 2.39 (− 11.11, 6.34)	− 8.74* (− 17.44, − 0.05)	7.07 (− 1.66, 15.79)
Stage N2 sleep time, min	5.73 (− 18.60, 30.07)	1.71 (− 22.40, 25.82)	− 15.95 (− 40.29, 8.38)
Total sleep time, min	13.55 (− 17.33, 44.43)	12.29 (− 18.34, 42.92)	− 34.48* (− 65.36, − 3.61)
Number of awakenings	− 0.11 (− 2.66, 2.44)	− 1.35 (− 3.88, 1.18)	2.28 (− 0.27, 4.83)
Wake after sleep onset, min	− 6.36 (− 37.54, 24.81)	− 22.45 (− 53.23, 8.33)	9.88 (− 21.30, 41.05)
Sleep efficiency, %	2.82 (− 3.6103, 9.2554)	2.56 (− 3.8219, 8.9420)	− 7.18* (− 13.6171, − 0.7514)

*P ≤ 0.10

*LSM* least squares mean, *PSG* polysomnography

### Quantitative EEG results

During REM sleep, 70-mg ASP8062 increased the absolute power of frequency bands between 1 and 12 Hz (delta, theta, and alpha bands) for the first third of the night and over the entire night and increased the absolute power of delta frequency bands for the second third of the night. Seventy milligrams ASP8062 also increased overall absolute power (1–48 Hz) for the first and second thirds of the night and over the entire night during REM sleep (Supplemental Table 1). In contrast, the relative power of waveforms > 12 Hz (sigma, beta, and gamma) was significantly decreased in each third of the night and over the entire night in the 70-mg ASP8062 group during REM sleep (Supplemental Table 2).

### Pharmacodynamics: change in GH

**Fig. 3:**
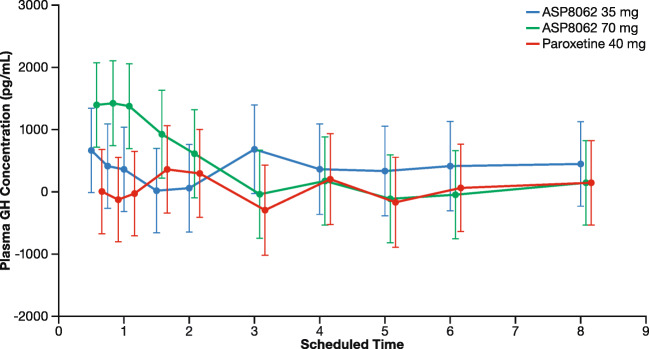
Linear scale plot of the change from baseline in plasma concentration of growth hormone LS means and 90% confidence interval following treatment. Following dosing and sleep onset, a numerically greater change in GH from baseline was noted in the 70 mg ASP8062 treatment group compared with the placebo and paroxetine groups during the first few hours after sleep onset. No apparent changes in GH were seen in the 35-mg ASP8062 and paroxetine 40-mg groups compared with placebo. Abbreviation: GH, growth hormone

Following dosing and sleep onset, a numerically greater change in GH from baseline was noted in the 70-mg ASP8062 treatment group compared with the placebo and paroxetine groups during the first few hours after sleep onset. No apparent changes in GH were seen in the 35-mg ASP8062 and paroxetine 40-mg groups compared with placebo **(**Fig. 3**)**.

### Pharmacokinetics: AUC_24_ and *C*_max_

In general, pharmacokinetics of ASP8062 taken following nighttime dosing were consistent with those reported in the healthy volunteer single ascending dose study where morning dosing was conducted (Walzer et al. 2020). As expected, the AUC_24_ for 70-mg ASP8062 was approximately double that of 35-mg ASP8062 (Table 5) and exposure following the 70-mg single dose was similar to the 30-mg once-daily dosing at steady state. *C*_max_ was slightly less than dose-proportional between 35-mg ASP8062 and 70-mg ASP8062.

**Table 5:** Plasma pharmacokinetic parameters for ASP8062 (PK analysis set)

Parameter	ASP8062 35 mg (*n* = 19)	ASP8062 70 mg (*n* = 20)
AUC_last_, h•ng/mL, mean (%CV)	1440 (21.7)	2670 (25.4)
AUC_10_, h•ng/mL, mean (%CV)	462 (25.1)	833 (30.7)
AUC_24_, h•ng/mL, mean (%CV)	865 (22.9)	1570 (27.3)
*C*_max_, ng/mL, mean (%CV)	100 (31.6)	165 (28.4)
*T*_max_, h, median (range)	2 (1.5–8.5)	2 (1.5–4.0)

*AUC*_*10*_ AUC from time 0 to 10 h postdose, *AUC*_*24*_ AUC from time 0 to 24 h postdose, *AUC*_*last*_ area under concentration-time curve (AUC) from time 0 to last measurable concentration, *C*_*max*_ maximum plasma concentration, *CV* coefficient of variation, *PK* pharmacokinetic, *T*_*max*_ time to maximum plasma concentration

### Safety

A total of 18 TEAEs were reported among 11 (55.0%) volunteers during the course of the study. These included two TEAEs in two (10.5%) volunteers who received placebo, two TEAEs in two (10.5%) volunteers who received 35-mg ASP8062, four TEAEs in three (15.0%) volunteers who received 70-mg ASP8062, and 10 TEAEs in five (26.3%) volunteers who received paroxetine 40 mg. The most commonly reported TEAE was nausea within the system organ class (SOC) gastrointestinal disorders (reported for four (21.1%) subjects after receiving paroxetine 40 mg (Table 6). The TEAEs were generally considered by the investigator to be mild in severity, with the exception of two cases of ear pain (70-mg ASP8062, *n* = 1; placebo, *n* = 1), which were moderate in severity and deemed unrelated to the study drug. AEs of potential interest related to abuse included the following: dizziness (reported for one (5.0%) subject after receiving 70-mg ASP8062 and for two (10.5%) subjects after receiving paroxetine 40 mg) and somnolence (reported for one (5.3%) subject after receiving paroxetine 40 mg). All AEs of potential interest recovered/resolved within the same day and were considered by the investigator to be mild in severity and did not require any treatment. Nine of the 18 TEAEs were considered drug-related by the investigator; these were two TEAEs in two (10.0%) subjects who received 70-mg ASP8062 and seven TEAEs in four (21.1%) subjects who received paroxetine 40 mg. ASP8062 treatment was not associated with cardiovascular or gastrointestinal AEs throughout the study. Additionally, no single event was reported that would suggest that ASP8062 has the potential effect of causing or triggering suicidal ideation or behavior. There were no deaths, serious AEs, or TEAEs leading to treatment withdrawal during this study.

**Table 6:** Incidence of TEAEs occurring in ≥ 2 healthy volunteers (safety analysis set)

medDRA V18.0 Preferred Term	Placebo (*n* = 19) *n* (%)	ASP8062 35 mg (*n* = 19) *n* (%)	ASP8062 70 mg (*n* = 20) *n* (%)	Paroxetine 40 mg (*n* = 19) *n* (%)
Headache	1 (5.3)	1 (5.3)	1 (5.0)	1 (5.3)
Nausea	0	0	0	4 (21.1)
Dizziness	0	0	1 (5.0)	2 (10.5)
Vomiting	0	0	0	2 (10.5)
Ear pain	1 (5.3)	0	1 (5.0)	0 (0.0)
Somnolence	0	0	0	1 (5.3)

*TEAE* treatment-emergent adverse event

## Discussion

The results from this phase 1, single-dose PSG study suggest that ASP8062 penetrates into the CNS and has pharmacological activity at the doses studied. The safety/tolerability, pharmacokinetic, and pharmacodynamic profiles of ASP8062 make this GABA_B_ receptor PAM acceptable for future clinical studies in disorders where GABA tone modulation are thought to play a role.

Modulation of time spent in SWS, as well as increases in EEG delta band power and stimulation of GH release, has previously been seen following GABA_B_ agonist dosing in preclinical rodent and human experimental studies (Van Cauter et al. 1997; Hodor et al. 2015). The current PSG study further supports the human translation of this pharmacology. Interestingly, contrary to preclinical findings (Murai et al. 2019) of ASP8062, no significant effect of ASP8062 on REM sleep was present at either dose of ASP8062 over the whole night of recording. Lack of translation of REM effects may be due to the compensation of decreased N1 stage sleep in humans instead of decreased REM sleep in rodents. Rodent sleep is primarily scored as two stages: non-REM and REM sleep. Thus, changes in N1-type sleep may have been masked by the overall effect on non-REM sleep in the rodent model. Alternatively, there may be differences in mechanisms of sleep architecture between species.

The increase in SWS and GH by ASP8062 primarily occurred during the first third of the night, a time during which pulsatile release of GH and SWS pressure is greatest. As ASP8062 has no GABA_B_ agonist properties itself, these data suggest that ASP8062 is increasing SWS and GH through modulation of the GABA_B_ receptor during times when endogenous GABA is being released—primarily early in the night (first third of the night). Following *T*_max_ in the CSF, concentrations of ASP8062 were still significantly elevated throughout the night (Walzer et al. 2020), which supports the notion that ASP8062 is likely modulating endogenous activation of the receptor. However, we cannot rule out the possibility that the effects seen during the first third of the night may be attributed to an absolute ASP8062 *C*_max_ concentration, which would primarily occur during this early time frame given the study design. Additional studies that correlate CSF concentrations with SWS and GH, and/or dose ASP8062 earlier or later in the night, are required to more fully understand these mechanisms. Interestingly, multiple clinical studies have confirmed that sodium oxybate, an agonist, also leads to increased SWS only during the early stages of sleep. However, it is unknown whether the lack of effects later in the night is due to less drive for SWS or the short half-life of sodium oxybate.

The current study included a healthy volunteer population and single-dose regimen for ease of signal detection. However, this design precluded an assessment of clinical significance. Study volunteers had normal sleep patterns and did not suffer from diseases often associated with abnormal sleep patterns. Similarly, these subjects would likely have normal GABA tone during periods of sleep. Although mean SWS only increased approximately 12 min (70-mg dose) over the night in a healthy volunteer population, additional clinical studies in a patient population with altered GABA tone during sleep, including those with narcolepsy, would be required to better assess the clinical significance of a GABA_B_ PAM as a therapeutic.

With regard to safety, ASP8062 was generally well tolerated. The majority of TEAEs in healthy volunteers who received ASP8062 were mild and unrelated to study treatment. Furthermore, ASP8062 treatment was not associated with cardiovascular or gastrointestinal AEs, and no events suggest ASP8062 has a potential triggering effect on suicidal ideation or behavior. These findings with this GABA_B_ receptor PAM are encouraging given that the development of baclofen and sodium oxybate has been limited by neurologic complications including sedation, coma, respiratory depression, and seizures (Russell et al. 2011; Dario and Tomei 2004; Chin et al. 1998; McCarson and Enna 2014). The potentially improved safety profile of ASP8062 compared with baclofen and sodium oxybate is likely explained by the fact that ASP8062 is a PAM of the GABA_B_ receptor (McCarson and Enna 2014) and therefore selectively binds to an allosteric site of the GABA_B_ receptor without binding and activating the receptor at the orthosteric site that binds GABA. Thus, ASP8062 enhances receptor activity with lower risk of tolerance issues and undesirable side effects compared with direct GABA_B_ agonists, baclofen, and sodium oxybate, both of which lack subtype selectivity.

Paroxetine was chosen as the positive control for this study based on its reduction in REM sleep, which has been assessed extensively in healthy volunteers and patients with depression, making it an ideal positive control for the current study (Winokur et al. 2001; Wilson et al. 2004; Barbanoj et al. 2005). The 40-mg dose of paroxetine was selected based on a review of the literature, which indicates a stable effect of paroxetine on sleep when the dosage is between 40 and 60 mg/day (Winokur et al. 2001). In the current study, the majority of TEAEs were reported for healthy volunteers receiving paroxetine; the most common TEAEs were nausea, vomiting, dizziness, and somnolence. While the safety profile of paroxetine 40 mg was as expected (Paxil [package insert] 2012), and can be attributed to the dose tested (40 mg), a lower dose of 20 mg may have been better tolerated.

In conclusion, ASP8062 is a GABA_B_ receptor PAM with an acceptable safety and tolerability profile and a favorable pharmacokinetic profile. Results from the current PSG study suggest that ASP8062 has pharmacodynamic effects in the brain as demonstrated by a significant and likely dose-related increase in SWS over the entire night, in conjunction with an increasing trend in GH. Furthermore, these data support the translatability between rodent and human experimental models of sleep with regard to assessment of GABA_B_-mediated pathways. The overall results suggest that ASP8062 modulates GABA_B_ receptors and improves endogenous sleep patterns. The current data support further development of ASP8062 for indications in which the GABA_B_ receptor pathway in the central nervous system is a target.

## Supplementary information

ESM 1 (DOCX 55 kb)

## Data Availability

Researchers may request access to anonymized participant level data, trial level data, and protocols from Astellas sponsored clinical trials at www.clinicalstudydatarequest.com. For the Astellas criteria on data sharing see: https://clinicalstudydatarequest.com/Study-Sponsors/Study-Sponsors-Astellas.aspx.

## Abbreviations

AEs: Adverse events
AUC_10_: AUC from the time of dosing to 10 h postdose
AUC_24_: AUC from the time of dosing to 24 h postdose
AUC_last_: Area under the concentration-time curve from the time of dosing to the last measurable concentration
*C*_max_: Maximum concentration
CSF: Cerebrospinal fluid
EEG: Electroencephalography
GABA: γ-Aminobutyric acid
GH: Growth hormone
PAM: Positive allosteric modulator
PDAS: Pharmacodynamic analysis set
PKAS: Pharmacokinetic analysis set
PSG: Polysomnography
PSGAS: PSG analysis set
REM: Rapid eye movement
SAF: Safety analysis set
SWS: Slow-wave sleep
TEAEs: Treatment-emergent adverse events
*T*_max_: Time of maximum concentration
