# Supplementary material for: A randomized phase 1 single-dose polysomnography study of ASP8062, a GABAB receptor positive allosteric modulator
Source: Psychopharmacology (Berl). 2021 Jan 12;238(3):867–76. doi: 10.1007/s00213-020-05738-y (PMC7914186; doi:10.1007/s00213-020-05738-y)
Supplement: Supplementary file 1 — (DOCX 55 kb) [file 213_2020_5738_MOESM1_ESM.docx]

**A Randomized Phase 1 Single-Dose Polysomnography Study of ASP8062, a GABA_B_ Receptor Positive Allosteric Modulator**

Mark Walzer^1^, Ruishan Wu^1^, Maha Ahmad^2^, Jon Freeman^2^, Gary Zammit^2^, Gerard J Marek^1^

^1^Astellas Pharma Global Development, Inc., Northbrook, USA; ^2^Clinilabs Drug Development Corporation, New York, USA

**Target Journal:** *Psychopharmacology*

**Corresponding Author:**

Mark Walzer
One Astellas Way

Northbrook, IL 60062

[mark.walzer@astellas.com](mailto:mark.walzer@astellas.com)

242-205-5102

**Supplemental Table 1. Average absolute power during slow-wave sleep (stage N3) and rapid eye movement sleep compared with placebo**

| **Absolute Power (LS Mean Difference)** | **Comparison versus Placebo** | | | | | | | | | | | |
| --- | --- | --- | --- | --- | --- | --- | --- | --- | --- | --- | --- | --- |
|  | **First Third of the Night** | | | **Second Third of the Night** | | | **Last Third of the Night** | | | **Whole Night** | | |
|  | **ASP8062** | | **Paroxetine** | **ASP8062** | | **Paroxetine** | **ASP8062** | | **Paroxetine** | **ASP8062** | | **Paroxetine** |
|  | **35 mg** | **70 mg** | **40 mg** | **35 mg** | **70 mg** | **40 mg** | **35 mg** | **70 mg** | **40 mg** | **35 mg** | **70 mg** | **40 mg** |
|  | **(n = 19)** | **(n = 20)** | **(n = 19)** | **(n = 19)** | **(n = 20)** | **(n = 19)** | **(n = 19)** | **(n = 20)** | **(n = 19)** | **(n = 19)** | **(n = 20)** | **(n = 19)** |
| **REM** | | | | | | | | | | | | |
| Delta (1 - 3.5 Hz) | 5.953 | 19.569** | 2.719 | 0.342 | 6.048* | 5.415 | -0.205 | 3.156 | 2.322 | 1.268 | 6.691** | 1.702 |
| Theta (3.5 - 8 Hz) | 0.003 | 7.693** | 0.542 | -0.639 | 2.429 | 1.121 | -0.779 | 0.795 | -0.183 | -0.14 | 2.822** | -0.244 |
| Alpha (8 - 12 Hz) | -0.124 | 2.813** | -0.116 | -0.121 | 1.195 | -0.116 | -0.369 | 0.758 | 0.144 | -0.09 | 1.536*** | -0.299 |
| Sigma (12 - 16 Hz) | 0.018 | 0.329 | 0.201 | -0.199 | 0.303 | 0.169 | -0.136 | 0.058 | -0.061 | -0.071 | 0.268 | 0.192 |
| Beta 1 (16 - 24 Hz) | -0.118 | 0.085 | 0.455 | -0.083 | 0.078 | -0.042 | -0.319 | -0.263 | -0.239 | -0.116 | 0.027 | -0.002 |
| Beta 2 (24 - 32 Hz) | 0.007 | -0.118 | 0.144 | -0.133 | -0.122 | 0.042 | -0.212 | -0.240* | -0.146 | -0.111 | -0.098 | -0.003 |
| Gamma (32 - 48 Hz) | 0.005 | -0.045 | 0.105 | -0.115 | -0.112 | 0.113 | -0.066 | -0.115* | 0.017 | -0.061 | -0.075 | 0.123 |
| Overall (1 - 48 Hz) | 5.725 | 30.816** | 4.712 | -1.022 | 9.873* | 6.656 | -2.033 | 4.13 | 1.703 | 0.683 | 11.215** | 1.42 |
| **SWS** | | | | | | | | | | | | |
| Delta (1 - 3.5 Hz) | -96.243 | -2.604 | -77.948 | -108.214 | -227.942* | -288.046** | 166.237 | -32.616 | -101.611 | -95.802 | -76.817 | -146.056* |
| Theta (3.5 - 8 Hz) | -2.314 | 8.804 | -4.772 | -10.458 | -11.142 | -22.265*** | 5.175 | -10.254 | -21.839 | -4.328 | 0.024 | -11.771** |
| Alpha (8 - 12 Hz) | -2.299 | -0.981 | -4.138 | -2.564 | -3.002 | -4.859** | -0.358 | -4.537 | -8.202* | -1.897 | -1.917 | -3.824** |
| Sigma (12 - 16 Hz) | -0.688 | -0.527 | -0.564 | -1.565 | -1.572* | -1.977** | -0.256 | -1.124 | -2.69 | -0.957 | -0.987 | -1.402* |
| Beta 1 (16 - 24 Hz) | -0.119 | 0.171 | -0.161 | -0.534 | -0.548 | -0.908* | -0.215 | -1.191 | -1.676 | -0.307 | -0.22 | -0.599 |
| Beta 2 (24 - 32 Hz) | -0.104 | 0.094 | 0.075 | -0.16 | -0.19 | -0.208 | -0.062 | -0.391 | -0.556 | -0.12 | -0.056 | -0.053 |
| Gamma (32 - 48 Hz) | -0.094 | 0.164 | 0.106 | -0.264 | -0.308 | -0.189 | -0.009 | -0.369 | -0.317 | -0.114 | -0.023 | 0.051 |
| Overall (1 - 48 Hz) | -101.696 | 5.372 | -87.404 | -123.111 | -244.436* | -318.553** | 169.526 | -53.221 | -139.402 | -103.388 | -79.822 | -163.652* |

All randomized subjects who received at least 1 dose of study drug (ASP8062, paroxetine or placebo) and had at least 1 polysomnography or electroencephalography result.

Average Absolute Power (uV2) during rapid eye movement sleep: mean of absolute powers of C3, C4, O1 and O2 electrodes. Average Absolute Power (uV2) during slow-wave sleep

(N3): mean of absolute powers of F3, F4, C3 and C4 electrodes.

n = the total number of subjects in the treatment group. The number of subjects included in the statistical analyses is available in End-of-Text Tables 12.5.2.1 and 12.5.2.2.

Nominal P value ≤ 0.1 based on 2-sided test without multiplicity adjustment was considered statistically significant.

P ≤ 0.10 = *; P ≤ 0.05 = **; P ≤ 0.01 = ***; P ≤ 0.001 = ****.

LS: least square; REM: rapid eye movement; SWS: slow-wave sleep.

**Supplemental Table 2. Average relative power during slow-wave sleep (stage N3) and rapid eye movement sleep compared with placebo**

|  | **Relative Power (LS Mean Difference)** | **Comparison versus Placebo** | | | | | | | | | | | |
| --- | --- | --- | --- | --- | --- | --- | --- | --- | --- | --- | --- | --- | --- |
|  |  | **First Third of the Night** | | | **Second Third of the Night** | | | **Last Third of the Night** | | | **Whole Night** | | |
|  |  | **ASP8062** | | **Paroxetine** | **ASP8062** | | **Paroxetine** | **ASP8062** | | **Paroxetine** | **ASP8062** | | **Paroxetine** |
|  |  | **35 mg** | **70 mg** | **40 mg** | **35 mg** | **70 mg** | **40 mg** | **35 mg** | **70 mg** | **40 mg** | **35 mg** | **70 mg** | **40 mg** |
|  |  | **(n = 19)** | **(n = 20)** | **(n = 19)** | **(n = 19)** | **(n = 20)** | **(n = 19)** | **(n = 19)** | **(n = 20)** | **(n = 19)** | **(n = 19)** | **(n = 20)** | **(n = 19)** |
|  | **REM** | | | | | | | | | | | | |
|  | Delta (1 - 3.5 Hz) | 3.413 | 3.52 | -2.537 | 2.215 | 2.837** | 5.390*** | 1.83 | 3.366** | 2.614 | 1.855 | 2.881** | 1.843 |
|  | Theta (3.5 - 8 Hz) | -1.665 | 1.015 | -0.945 | -0.309 | 0.575 | -1.2 | -0.335 | -0.29 | -1.126 | -0.271 | 0.347 | -1.106* |
|  | Alpha (8 - 12 Hz) | -0.064 | -0.089 | 0.486 | -0.405 | -0.604 | -2.622*** | -0.3 | -0.111 | -0.442 | -0.264 | -0.181 | -1.165 |
|  | Sigma (12 - 16 Hz) | -0.314 | -1.309** | 1.076 | -0.392 | -0.554* | -0.621 | -0.21 | -0.583* | -0.212 | -0.264 | -0.694 | 0.296 |
|  | Beta 1 (16 - 24 Hz) | -0.738 | -1.722*** | 0.964 | -0.252 | -0.969**** | -0.640** | -0.551* | -1.178 | -0.515 | -0.467* | -1.182 | -0.068 |
|  | Beta 2 (24 - 32 Hz) | -0.343 | -1.010*** | 0.035 | -0.368* | -0.739**** | -0.255 | -0.255 | -0.676 | -0.198 | -0.331* | -0.742 | -0.006 |
|  | Gamma (32 - 48 Hz) | -0.2 | -0.559** | 0.15 | -0.39 | -0.548** | -0.017 | -0.144 | -0.464 | -0.079 | -0.27 | -0.509 | 0.149 |
|  | **SWS** | | | | | | | | | | | | |
|  | Delta (1 - 3.5 Hz) | -0.657 | 0.484 | -0.643 | -0.165 | -0.542 | -1.778 | 7.769* | 7.755* | 4.837 | 0.021 | 0.928 | -0.181 |
|  | Theta (3.5 - 8 Hz) | 0.594 | 0.256 | 0.362 | -0.057 | 0.336 | 0.471 | -4.274* | -4.395* | -3.216 | 0.126 | -0.238 | -0.143 |
|  | Alpha (8 - 12 Hz) | 0.025 | -0.483* | -0.014 | -0.007 | 0.024 | 0.613* | -2.397* | -2.367* | -1.443 | -0.138 | -0.446 | 0.033 |
|  | Sigma (12 - 16 Hz) | -0.031 | -0.226** | 0.112 | -0.001 | -0.014 | 0.281* | -0.483 | -0.369 | -0.109 | -0.06 | -0.193* | 0.108 |
|  | Beta 1 (16 - 24 Hz) | 0.025 | -0.056 | 0.06 | 0.066 | 0.075 | 0.183** | -0.344 | -0.379 | -0.144 | -0.01 | -0.067 | 0.042 |
|  | Beta 2 (24 - 32 Hz) | 0.015 | -0.01 | 0.058* | 0.063 | 0.064 | 0.121*** | -0.134 | -0.142 | -0.035 | 0.012 | -0.012 | 0.067** |
|  | Gamma (32 - 48 Hz) | 0.018 | 0.011 | 0.062* | 0.038 | 0.041 | 0.125*** | -0.116 | -0.129 | 0.033 | 0.015 | 0.001 | 0.100*** |

All randomized subjects who received at least 1 dose of study drug (ASP8062, paroxetine or placebo) and had at least 1 polysomnography or electroencephalography result.

Average Relative Power (%) during rapid eye movement sleep: mean of relative powers of C3, C4, O1 and O2 electrodes. Average Relative Power (%) during slow-wave sleep (N3):

mean of relative powers of F3, F4, C3 and C4 electrodes. Relative power was derived as the absolute power in predefined frequency band divided by the absolute power in overall

band for a particular electrode, multiplied by 100.

n = the total number of subjects in the treatment group. The number of subjects included in the statistical analyses is available in End-of-Text Tables 12.5.2.3 and 12.5.2.4.

Nominal P value ≤ 0.1 based on 2-sided test without multiplicity adjustment was considered statistically significant.

P ≤ 0.10 = *; P ≤ 0.05 = **; P ≤ 0.01 = ***; P ≤ 0.001 = ****.

LS: least square; REM: rapid eye movement; SWS: slow-wave sleep.
